# Research on Ecological Landscape Design and Healing Effect Based on 3D Roaming Technology

**DOI:** 10.3390/ijerph191811406

**Published:** 2022-09-10

**Authors:** Zhengsong Lin, Yuting Wang, Yang Song, Tao Huang, Feng Gan, Xinyue Ye

**Affiliations:** 1 Virtual Landscape Design Lab, School of Art and Design, Wuhan Institute of Technology, Wuhan 430205, China; 2 Tus-Design Group Co., Ltd., Suzhou 215000, China; 3 Department of Landscape Architecture and Urban Planning, Texas A & M University, College Station, TX 77840, USA; 4 School of Art, Culture and Tourism Industry Think Tank Chinese Art Evaluation Institute, Southeast University, Nanjing 211189, China

**Keywords:** sub-health, 3D roaming technology, ecological landscape, healing effect

## Abstract

Impacted by the COVID-19 epidemic, the human sub-health in national high-tech zones (hereinafter referred to as high-tech zones) has become more prominent. It is critical for the mental sub-health group in the high-tech zone to relieve the anxiety and tension caused by the pressure of life and work. This paper uses SketchUp virtual engine (Unity 2019) software, and 3D roaming technology to carry out the ecological landscape transformation design of the Baotzixi ecological corridor in the East Lake High-tech Zone, to construct a 3D roaming landscape scene and measure its therapeutic effect by inviting subjects to participate in an interactive experience experiment on the ErgoLAB platform. The results illustrate that: (1) the thermogram trend shows that the more attractive the 3D roaming landscape scene is, the stronger the subjects’ interest is; (2) the participants have a positive emotional arousal state in the immersive experience of the 3D roaming landscape scene after the modification design; and (3) the mean skin conductance (SC) fluctuation variance of the subjects is 5.819%, indicating that the healing effect is significant in the state of positive emotional arousal. The research results show that there is a connection between the subjects and the 3D roaming landscape scene after the transformation design of “high interest, emotional arousal and significant healing”.

## 1. Introduction

High-tech zones have become a major engine to promote innovative development in China. The sub-health problem among people in the high-tech area has increased during intense COVID-19 prevention and control. The overall incidence of sub-health in urban residents of six provinces and cities in China was 68.06%, the incidence of mental sub-health was 65.96%, and the incidence of social sub-health was 70.76% [1]. About 200–300 million people in China suffer from mental health problems, among which depression and anxiety rank first [2]. The basic characteristics of sub-health are that there is no obvious disease in the body, but the physical strength is reduced, the reaction ability is decreased, and the mental state is poor [3]. How to reconstruct the ecological landscape design of the research area, promote the mental sub-health groups in the high-tech zone, and relieve the anxiety and tension in the work pressure has become a key issue to be urgently solved. Healing landscapes utilize various environmental elements (e.g., plants, water bodies and animals) and landscape reconstruction design, so the human body can be stimulated to soothe itself and promote physical and mental health [4,5,6].

Through the permutation and combination of multiple natural factors, the healing landscape design builds an environment for people to relax their body and mind. Attention restoration theory (ART) states that directed attention is critical for human health [7]. Admiring natural landscapes can improve people’s directed attention to relieve stress. The biophilia hypothesis argues that human beings have an inherent tendency to get close to nature, which is rooted in the survival instinct in human evolution [8]. Failure to follow this tendency will cause the unbalance of physical and mental structure. The higher the urbanization level of people’s living areas, the higher their preference for natural landscape. Huisman et al. (2012) and Stichler (2001) proposed stress reduction theory (SRT). They pointed out that when individuals are in a state of stress, access to a certain natural environment can relieve the physiological, psychological, and behavioral harm caused by stress [9,10]. Natural scenery can stimulate positive emotions and then decrease the pressure. Kalantari (2014) advocated the idea that long-term exposure to green fields and natural scenery of flowers and trees can adjust moods [11].

Hu et al. (2017) believe that the advantage of virtual reality technology lies in the creation of virtual environment. When users are immersed in a virtual environment, the sensory experience is real, which is not inconsistent with the real environment experience, and is of great significance to promote the wide application of virtual reality and improve user experience [12]. Landscape settings can be made into 3D roaming landscapes through Sketch UP and Unity software to achieve an immersive experience [13,14,15]. The concept of metaverse has been increasingly popular [16,17,18,19]. Xu et al. (2020) studied the healing of urban environment through two VR experiments and integrated VR technology into the healing landscape [20]. NIKI et al. (2019) used skin electrical activity, heart rate and skin temperature to assess the symptoms of anxiety, depression, and pain in people with a history of sub-health diseases, and the results showed that participants’ anxiety and depression were more significantly improved [21]. MOSCATO et al. (2021), KABIR et al. (2020) and Wu et al. (2022) believe that virtual tourism based on VR technology can effectively reduce patients’ anxiety and depression and improve the negative emotions of cancer patients [22,23,24]. Palomba et al. (2000) found that when subjects were shown videos of different quality, the subjects’ autonomic nervous responses changed. When subjects watched videos of low quality, the skin conductance response increased, and the typical characteristics of stress response appeared, inducing negative emotional experience of the subjects [25]. Ward et al. (2003) asked the subjects to browse two types of web pages that followed or violated the design principles, and to complete the follow-up survey. The results showed that when the subjects browse the web pages that did not follow the design principles, the task performance was significantly reduced [26]. Drachen et al. (2011) found that heart rate and electrodermal response were negatively correlated with subjects’ subjective score of positive emotion in the game, that is, the lower the Heart rate of skin conductance wave value and electrodermal activity were, the higher the subjects’ sense of immersion, fluency, and positive emotion in the game [27]. Lin et al. (2022) and Stenling et al. (2018) used VR technology as an intermediary to study the healing urban environment through two VR experiments, and integrated VR technology into the healing landscape [28,29]. Although the application of VR and dermato-electric technology is gradually becoming popular, the above research does not combine 3D roaming with the user experience of sub-health groups to monitor the psychological perception effect of sub-health groups in real time. In December 2021, the Chinese government issued the “14th Five-Year” Digital Economy Development Plan to encourage the development of digital technology and accelerate the integration of various industries, and promote the upgrading and healthy development of the digital economy structure [30].

Relevant research in the literature has found that although the theoretical framework of healing landscape has been established, such as the “attention recovery theory” and the “biophilia hypothesis” mentioned above, the relationship between humans and nature is still more involved. The theoretical and practical research on the healing effect of 3D roaming technology is still insufficient. In terms of research methods, most existing studies use interview, questionnaire, and instrument measurement of objective data to obtain qualitative or quantitative research results, but such methods are often accompanied by many uncontrollable subjective factors. With the rise and popularity of metaverse technology, 3D roaming technology can be introduced into the research of healing architecture and healing landscape. Although some researchers have tried to use the method of VR + healing for research, the scope involved is relatively limited. The therapeutic effect of VR + healing on people is only analyzed in the aspects of buildings, streets and negative emotion management of cancer patients [20,24,31]. The healing effect is not explored from different landscape spatial relationships, and the virtual engine technology is not combined to provide better immersive experience from the interactive dimension of “human-machine-environment” to enrich the experience methods and effects of different people. In terms of policy implementation, local digital infrastructure has not yet been established and improved, and the digital application of public services is weak. There is a long way to go for digital transformation and upgrading.

In order to construct a 3D roaming landscape scene with a healing effect on sub-health people and relieve pressure, this paper intends to put forward a “digital roaming landscape design–arousal–healing effect evaluation” innovation model. Using Unity2019-derived virtual roaming, VR headsets and skin conductance were worn after the renovation design of ecological landscape scenes. The immersive experience experiment of a virtual roaming scene was conducted, and the ErgoLAB platform was used to record data synchronously. After the experiment, the participants were invited to conduct a questionnaire survey on the 3D landscape roaming experience, so as to provide effective measures for alleviating the psychological sub-health symptoms of the national high-tech zone.

## 2. Materials and Methods

### 2.1. Study Area

**Figure 1:**
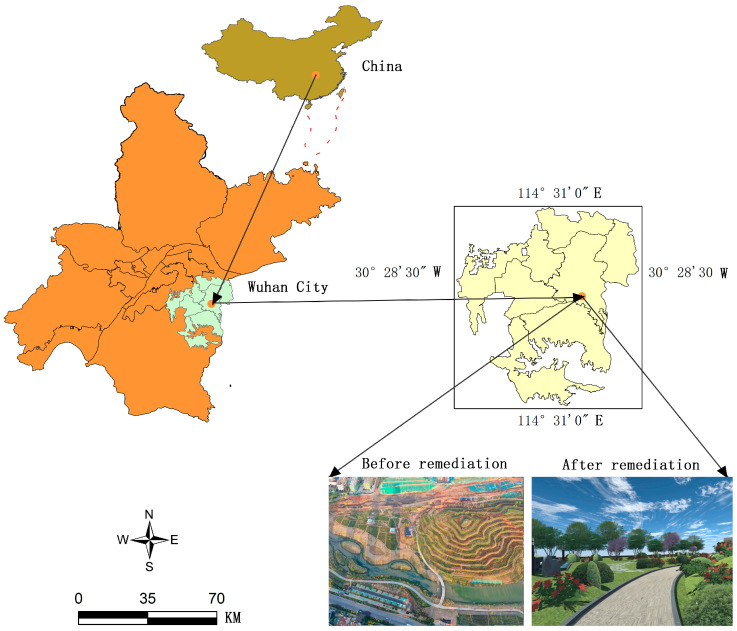
Location overview diagram of Baozixi eco-corridor in the high-tech zone. source: shot by DJI inspire 2 (DJI Technology Co., Ltd., Shenzhen, China).

Located in the southeast of Wuhan city, East Lake High-tech Zone is a national opto-electronics industry base and pilot free trade zone of China, with a planned area of 518 square kilometers and a number of universities, colleges and provincial and ministerial research institutes gathering here. Baozixi Ecological Corridor is the core area of East Lake High-tech Zone, located on the south of Gaoxin Avenue, east of Optics Valley 4th Road, west of Baoxi Road, and north of Gaoxin 5th Road (30°45′48″ N–111°49′42″ E), with a total area of about 1199 hectares and a total length of 5.090 km^2^, as shown in Figure 1.

With the environmental pollution caused by the expansion of high-tech zones as well as the strict COVID-19 prevention and control in China, the R and D personnel are apt to have anxiety, depression, and tension [32]. There is a sharp increase in the risk of people with mental sub-health transitioning to physical sub-health [33,34,35]. At present, the flower vegetation patches in the ecological corridor are unevenly distributed with limited varieties. Seasonal factors are not considered, leaving a large amount of vegetation and flowers withered. The aesthetic and therapeutic properties of the landscape are inadequate to address the healing needs of local residents. Thus, it is urgent to reform and redesign the ecological corridor.

In order to study the effect of a 3D roaming landscape designed after ecological landscape transformation on emotional arousal state and healing of mental sub-health groups, the VR eye movement, electrodermal activities (EDA) skin electrical test, control questionnaire and positive and negative emotion adjective scale were combined to carry out the research of landscape healing and the following hypotheses were proposed:

(I) There is a correlation between the subjects’ interest in 3D roaming landscape and their emotional arousal.

(II) After the immersion experience, the lower the SC variance value of positive emotional state, the more significant the healing effect.

### 2.2. Quantity Collection and Processing

#### 2.2.1. Data Collection

We used DJI Inspire 2 and TITAN360 panoramic camera (Shenzhen LAN Feng Chuangshi Network Technology Co., Ltd., Shenzhen, China) to collect data such as topography, vegetation, flowers, and buildings. Second, 200 questionnaires with a history of mental sub-health diseases were collected as experimental subjects. The healing perception evaluation of the participants on the existing landscape scene was collected, which was used for the control verification of the landscape scene after the ecological landscape transformation design. A total of 200 questionnaires with a history of mental sub-health diseases were collected as experimental subjects.

#### 2.2.2. Scene Construction

Based on the theory of landscape ecology, the virtual landscape scene was built in SketchUp Export using the FBX format and imported into Unity 2019 to set the VR-Plugin program. We added Box Collider collision so that the eye tracker can detect the object and record the eye movement data. The position of the camera rig helmet was adjusted to make the eye level height consistent with the eye level height of the subject. The 3D roaming landscape package was exported and connected to the ErgoLAB platform. The participants wore helmets and walked with the handle to have an immersive experience.

#### 2.2.3. VR and Skin Electricity Experiment 

**Figure 2:**
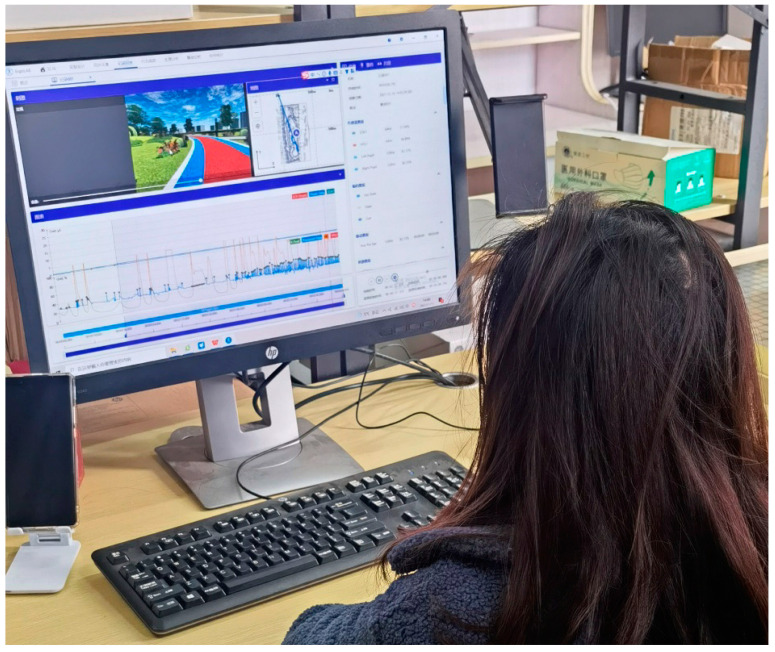
Experimental scene.

**Figure 3:**
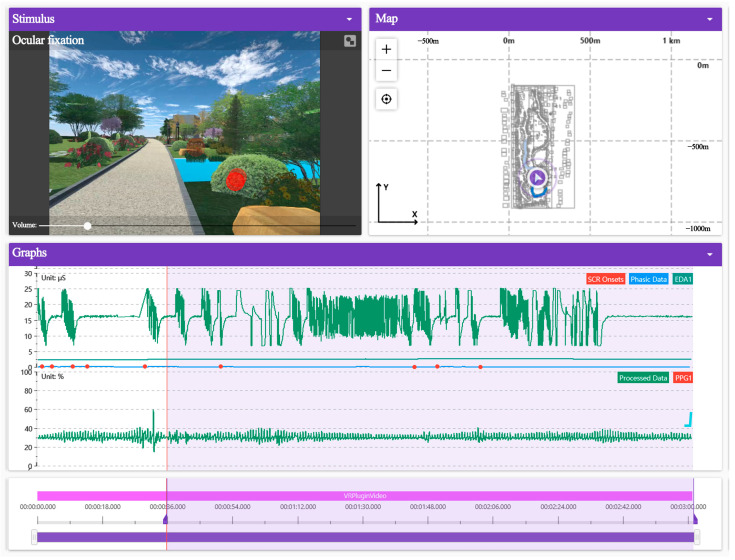
Experimental scene and data monitoring interface. Source: ErgoLAB platform experiment export.

This experiment adopted a 2-factor (high/low interest) × 2-level (positive/negative emotion) inter-subject design, and selected subjects’ high/low interest in 3D roaming landscape and positive/negative emotion states as independent variables, and selected with/without healing effect as dependent variables. The principal of the experiment organized the subjects to become familiar with the experiment environment, process and instruction to ensure the accuracy and reliability of the experiment. The experiment was divided into three stages: the pre-test stage was the experimental calibration. In the 200 questionnaires about the history of mental sub-health diseases, 80 people (including researchers from Xiaomi and Huawei, designers from China Construction Third Engineering Bureau and researchers from universities, etc.) were randomly invited, (including 52 males and 28 females, with naked eyes ≥ 1.0). Calibration on ErgoLAB platform was conducted to ensure that the error of five line of sight points was no more than 20 pixels. The skin electrical fluctuation frequency should be normal with a sampling rate of 120 Hz. In the experimental stage, the subjects conducted virtual immersive roaming in the scene according to the instructions to experience highly realistic human–computer interaction. The subjects could choose the roaming route, move freely and observe the landscape according to their own wishes. The above steps were repeated until 80 subjects completed the experiment, as shown in Figure 2 and Figure 3. Post-test stage: first, according to Watson et al. 1988, the positive and negative affect scale (PANAS) was proposed to conduct the emotional arousal test [36,37]. The experimenter invited the subjects to complete a subjective questionnaire in order to verify the reliability of the healing effect. The content included positive and negative emotional adjective scales, namely positive emotional adjective (PA) and negative emotional adjective (NA), giving corresponding emotional matching measurement values (5 points—very many; 4 points—more; 3 points—medium level; 2 points—a little; 1 point—very little or almost none) to evaluate the positive and negative emotions of the participants after the immersive experience. Second, the experimental and questionnaire data were exported. At the same time, the integrity of the experimental data was observed, and 73 valid samples were obtained by eliminating 7 samples with large error, with an effective sample rate of 91.25%. 

### 2.3. Data Visualization

Firstly, the subjects’ interest in the experimental scene was calculated, and the hotspot map of the landscape category was derived and analyzed to help verify whether the subjects experienced high interest after the immersive experience. Secondly, the correlation analysis between gaze frequency and SC wave value was conducted to analyze the relationship between subjects’ interest in the four types of landscapes and emotional arousal. Finally, the significance of the healing effect was verified by combining experimental results, support from the literature and the subjective questionnaire, so as to form a logical relationship between interest experience, emotional arousal and healing effect:

#### 2.3.1. Visualization of Interest Experience 

After a 3-min immersive experience in the roaming landscape scene, the experimental data were extracted and normalized by ErgoLAB platform. In order to confirm that the 3D roaming scene after ecological landscape design can stimulate the change of interest degree of the subjects, the degree of interest is measured by the weight of the subjects’ immersive experience. The larger the weight, the stronger the interest intensity of the roaming scene. In this paper, the degree of interest was calculated according to the fixation time, fixation times and pupil diameter of the participants in the immersive experience [38], namely:

(1) $$\begin{array}{l} Y_{ij}=\frac{\max x_{ij}-x_{ij}}{\max x_{ij}-x_{ij}};i\in \left[1,m\right],j\in \left[1,n\right]\&\\ Y_{ij}=\frac{x_{ij}-\min x_{ij}}{\max x_{ij}-\min x_{ij}};i\in \left[1,m\right],j\in \left[1,n\right] \end{array}$$

(2) $$f_{ij}=Y_{ij}/\sum_{j=1}^{n}Y_{ij}$$

(3) $$I_{i}=\frac{t_{i}}{\sum_{i=1}^{n}t_{ij}}+\frac{c_{i}}{\sum_{i=1}^{n}c_{ij}}+\frac{p_{i}}{\sum_{i=1}^{n}p_{ij}}$$

where *Y_ij_* is the normalized value, *x_ij_* is the value from *i* to *j*, *max_ij_* and *min_ij_* are the maximum and minimum values from *i* to *j*, respectively. *I_i_* is the ith degree of interest, *t_ij_* is the time from the *i*th fixation point to the *j*th fixation point, *C_ij_* is the number from the ith fixation point to the *j*th fixation point, *P_ij_* is the pupil diameter from the *i*th fixation point to the *j*th fixation point, and *n* is the total number of fixation points.

#### 2.3.2. Correlation Analysis of Interest and Emotional Arousal

To investigate the relationship between the subjects’ interest and emotional arousal, a correlation analysis was conducted between gaze frequency and SC wave value. We observed whether there was a significant correlation between interest and emotional arousal state. Combined with the data of the positive and negative emotion adjective scale, we verified the positive and negative emotion arousal state of the participants after immersive experience.

#### 2.3.3. Analysis of Healing Effect

**Figure 4:**
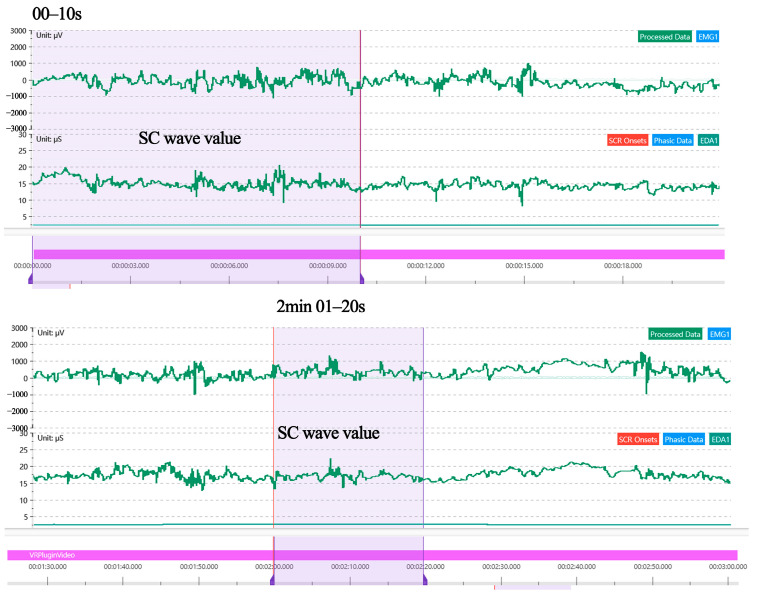
The skin conductance wave value response. Source: The emotional arousal state between 0–10 s and 1–20 s of 2 min was intercepted to calculate the skin conductance range of the subjects.

The skin resistance and conductance of the human body change with the function of skin sweat glands. These measurable skin electrical changes are called electrodermal activity, which consists of skin conductance, skin conductance level and skin conductance response. Skin conductance is the most sensitive index to evaluate the level of emotional arousal, which can reflect the overall response and degree of people in psychological and physiological states [39]. In the state of emotional arousal, sweat gland secretion is affected by emotional state. When people are in a tense mood, sweat secretion increases, and the resistance of trace current passing through sweat becomes smaller, and skin conductance increases. When a person is in a relaxed mood, skin conductance levels decrease [40]. Therefore, the resistance encountered by a small amount of electric current passing through the skin can be used to measure the emotional response of the autonomic nervous system. There are significant changes in skin electrical signals with different emotions. We calculate the variance of skin conductance output value in two-time sections as baseline, between 2 min 1–20 s and 0–10 s (baseline). These values, called skin conductance wave values, were used to reflect the subjects’ emotions and arousal states as shown in Figure 4.

According to Ward et al. (2002), when the subject pressure increased, the skin conductance variance showed an upward trend [26]. The skin conductance fluctuation was used to reflect the emotional state of the subjects. When the skin conductance wave value was <7%, it indicated that the subjects were in a low-pressure environment and experienced calm and soothing emotions, which could produce therapeutic effects; when the skin conductance wave value was ≥7%, it indicated that the subjects were in a high-pressure environment and experienced emotional tension. In this paper, the SC fluctuation variance value is used to reflect the healing effect of the subjects. When SC is <7%, it indicates that the subjects are in a state of low pressure, calm and soothing, and can produce healing effects. When SC is ≥7%, it indicates that the subjects are in a state of high stress and emotional tension, and the healing effect is not significant.

### 2.4. Technical Framework Diagram

**Figure 5:**
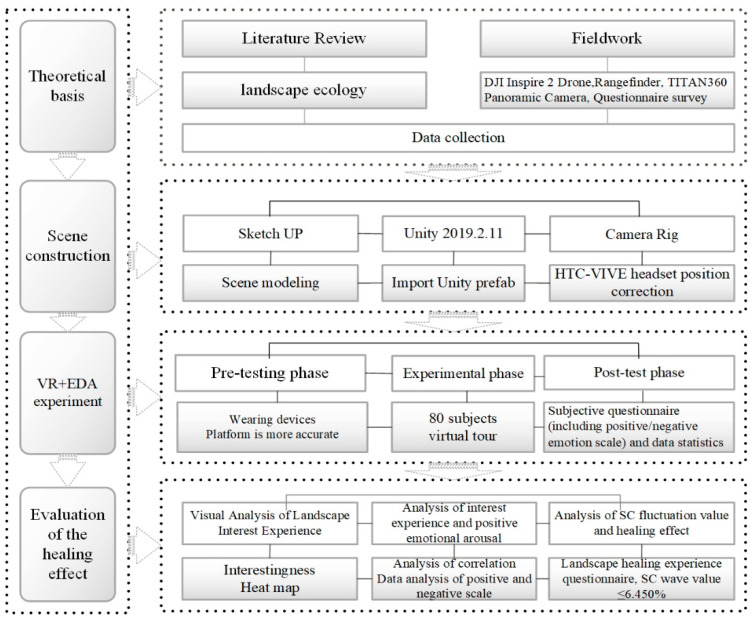
Research framework diagram.

The technical framework of this paper presents a logical flow of theoretical preparation–scene construction–VR and EDA experiment–healing test, as shown in Figure 5.

Firstly, through the literature review, relevant landscape ecology theories are summarized. Based on field investigation, DJI Inspire 2 and TITAN360 panoramic camera were used to collect data. Secondly, the 3D roaming landscape scene was constructed through SketchUp and Unity2019.2.11, and subjects were invited to conduct VR and EDA experiments. ErgoLAB platform recorded the experimental data of subjects. In the post-test phase, subjects were invited to fill in subjective questionnaires. Finally, SPSS software was used to analyze the subjective and objective data of the experiment to verify the feasibility of the therapeutic effect.

## 3. Results

In order to verify the landscape healing effect of 3D roaming immersive experience after ecological landscape transformation design, and subjects with a history of mental sub-health diseases were randomly invited to participate in a 3D landscape roaming experience on the ErgoLAB platform. The eye movement hot map was observed to calculate the interest degree of the subjects, and the landscape category of interest and the interest degree value of the scene were obtained. The correlation between the gaze frequency of the four types of landscapes and SC was analyzed, and the emotional arousal state of the subjects was analyzed with the positive and negative emotional adjective scales. Through the experimental data, questionnaire data and the literature, the significance of the healing effect of the participants after the immersive experience was confirmed, presenting the logical relationship of “interest experience–emotion change–therapeutic effect”.

### 3.1. The Change Trend of Interest and Heat Map Confirmed That the Subjects Experienced High Interest in the Immersive Experience

**Figure 6:**
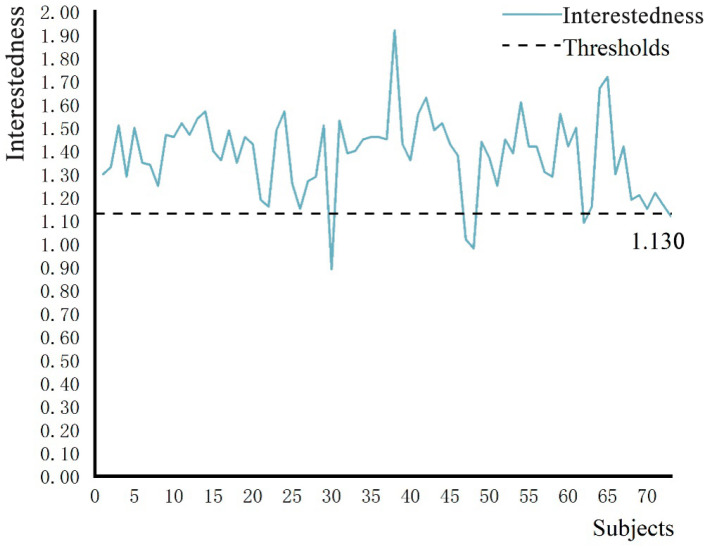
Variation trend of subjects’ level of interest. Source: ErgoLAB platform derived the data, and the weight threshold of the level of interest was 1.130 when the time of the fixation point was the longest.

**Figure 7:**
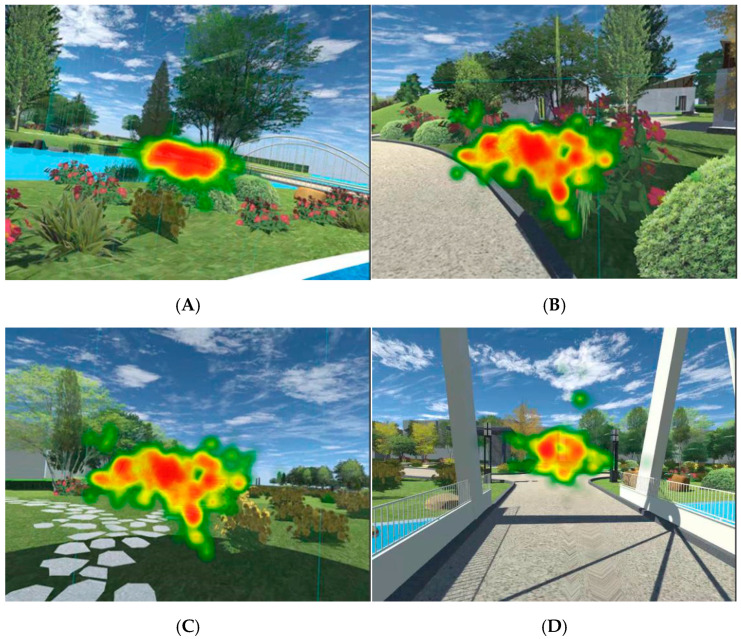
Heat map of eye movement and fixation changes in the region of interest. Source: ErgoLAB platform export. (**A**) Vegetation landscape interest area and heat map; (**B**) flower landscape heat map; (**C**) heat map of interest area of road landscape; (**D**) heat map of architectural landscape interest area.

In order to ascertain the subjects’ interest in the 3D roaming landscape scene, fixation duration, fixation frequency and pupil diameter were used to calculate subjects’ level of interest in roaming scenes. According to Formulas (1)–(3), the changing trend of level of interests of 73 subjects was obtained, as shown in Figure 6. Heat maps of four types of landscape nodes of the subjects were derived through the ErgoLAB platform, as shown in Figure 7.

The results show that when the interest degree of the subjects after the immersion experience was ≥1.130, the subjects have high interest in the 3D roaming landscape scene after the transformation design. As visualized in Figure 6, 68 subjects in the roaming landscape scene had an interest degree ≥ 1.130, accounting for 93.15% of the total number. There were five subjects whose interest degree was <1.130, accounting for only 6.85% of the total number, indicating that subjects experienced strong interest in the 3D roaming landscape scenes, which was consistent with the research results of Wang et al. (2016). As visualized in Figure 7, the stronger the attraction of the 3D roaming scene, the higher the subjects’ interest and the deeper the color concentration; the less attractive the 3D roaming scene was, the lower the subjects’ interest was and the lighter the color concentration. The research results confirm the change trend of the degree of interest, and the heat map confirms that the subjects have a high degree of interest in the 3D roaming landscape scene after the transformation design.

### 3.2. Positive Emotional Arousal State of Subjects after Immersive Experience of 3D Roaming Landscape Scene

**Figure 8:**
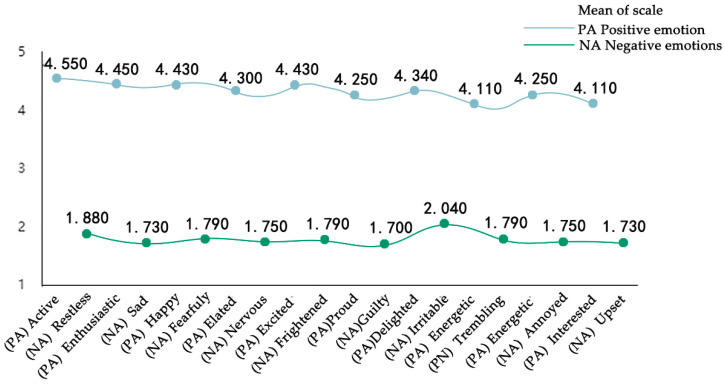
Trend of positive and negative emotion scales.

In order to investigate whether there is a significant correlation between level of interest and positive emotion arousal in 3D roaming landscape scenes, this paper analyzed the correlation between vegetation landscape, flower landscape, architectural landscape and other landscapes and SC, respectively, as shown in Table 1. The results showed that skin conductance was significantly correlated with flower landscape (*r* = 0.008, *p* = 0.050) and vegetation landscape (*r* = 0.038, *p* = 0.050), respectively. There was no significant correlation between SC and architectural landscape (*r* = 0.298, *p* = 0.050) and other landscapes (*r* = 0.729, *p* = 0.050). The research results showed that the subjects’ interest in the 3D roaming landscape was correlated with their emotional arousal. According to the positive and negative emotional adjective scales (*PA* = 43.310, *NA* = 17.950) PA > NA in Figure 8, after the modified 3D roaming immersive experience, the participants showed stable emotions, low psychological cognitive load and positive emotional arousal state.

### 3.3. The SC Fluctuation Variance of the Subjects Was Low after the Immersion Experience, Which Confirmed That the Healing Effect Was Significant

**Figure 9:**
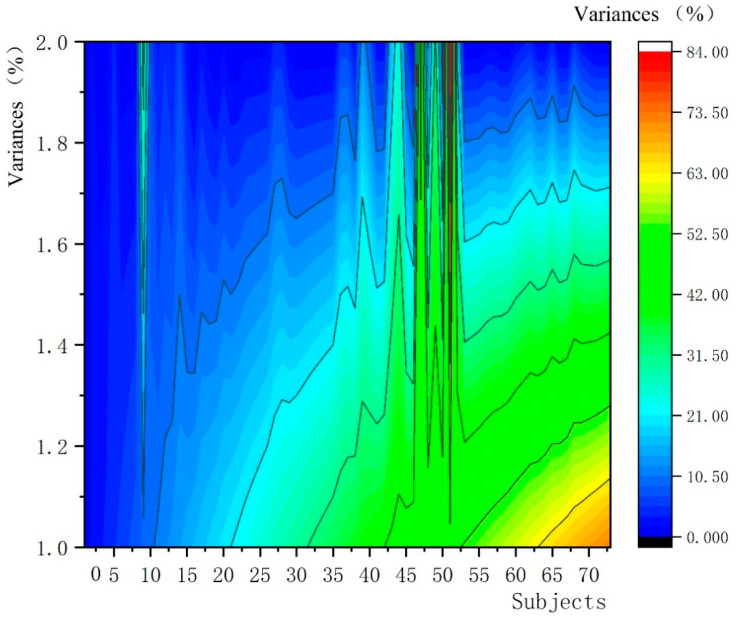
Trend chart of skin conductance wave value variance of subjects. Source: ErgoLAB platform derived the data and calculated that the minimum variance of skin conductance was 0, the maximum was 84%, and the average wave value was 5.819%.

In this paper, visual analysis was conducted on skin conductance fluctuation variance in EDA, as shown in Figure 9. The average wave value of skin conductance fluctuation variance of 73 subjects was calculated to be 5.819%. Among them, there were 63 subjects whose skin conductance fluctuation variance was smaller than 6%, accounting for 86.30% of the total number of subjects, and 51 subjects whose skin conductance fluctuation variance was ≤3%, accounting for 69.86% of the total number of subjects. Only 10 subjects’ skin conductance fluctuation variance values are larger than 6%, accounting for only 13.70% of all participants. The research results confirmed that after the immersive experience of the 3D roaming landscape scene, the level of emotional arousal and SC fluctuation variance was low, the level of stress and psychological cognitive load were low, and the healing effect was significant. The results are consistent with experimental Hypothesis 2. In order to further prove that the participants experienced therapeutic effects in the immersive experience, the field survey and immersive experience after statistical analysis of the questionnaire data were used for comparison with the statistical analysis. The results showed that before the ecological landscape design, the proportion of subjects with a healing effect response was only 35.28%, the reliability and validity test Cronbach coefficient (*α*) = 0.720, *KMO* = 0.758; after modification design and immersion experience, the proportion of subjects with a healing effect response was as high as 85.72%, Cronbach coefficient (*α*) = 0.631, *KMO* = 0.675. The subjective questionnaire results were statistically significant (P and LT; 0.001), indicating the reliability and construct validity of the subjective questionnaire. In conclusion, the research results show that the participants’ immersive experiences after ecological landscape design have low SC fluctuation variance and significant healing effect, which proves that the modified 3D roaming landscape scene can produce healing effects for people with mental sub-health, presenting the logical relationship of “interest experience, emotional arousal and healing effect”.

## 4. Conclusions

Research result 3.1 confirmed that the higher the fixation frequency, the darker the color concentration of the heat map, which proves that the subjects are more interested in the roaming landscape. This is consistent with the research results of Liu et al. (2021), Pei et al. (2021) and Lin et al. (2022) [41,42,43]. The variation trends of interest and thermodynamic diagram confirm that subjects have a strong interest in immersive experience, which is consistent with the research results of Lin et al. (2022) and Wang et al. (2016) [38,43]. Water landscape is not selected in the construction of the 3D roaming landscape. On the one hand, due to technical limitations, water flow needs to be integrated across disciplines to achieve realistic effects. On the other hand, if 3D water bodies are set in the roaming landscape scene, the number of faces in the FBX program is up to 3.5 million, exceeding the official threshold of Unity2019 (≤1.5 million). Most environmental elements we included were found to be important to user experiences in previous empirical studies [44,45,46]. In terms of the color of flower landscapes, the preliminary field study found that yellow, orange and yellow-green patches had better healing effects, which is consistent with the research results of Zhang et al. (2021) on the effect of plant color on visual fatigue [47]. Osmanthus, Chinese rose, gerbera, freesia, gardenia and other varieties were mainly selected for plant landscape, with appropriate distribution proportion of arbor, shrub and grass to maximize the therapeutic benefits, which is consistent with the research results of Chen et al. (2020) and Li et al. (2017) on plant landscape healing [48,49].

Research result 3.2 confirmed the positive emotional arousal state of the participants in the immersion of the 3D roaming landscape scene after ecological landscape design. In the positive and negative emotional adjective scales, gender differences exist in positive and negative emotional states in different regions. The research results of Schimmack et al. (2001) and Wang Li et al. (2007) found that there was no significant gender difference between men and women in Asia [50,51]. According to the assessment theory of emotional excitement, when external stimuli are rated as “favorable”, positive emotions will be generated; if they are rated as “harmful”, negative emotions will be generated [52,53]. The stimulation of a 3D roaming landscape scene itself is not “offensive” and “harmful”, so it can arouse the positive emotion of the subject. At this time, the psychological cognitive load of the subject is low, and the emotion is gentle. This is consistent with the research findings of Gouizi et al. (2011), Balconi et al. (2012) and Canavesio et al. (2013) [54,55,56].

Research result 3.3 confirmed that the psychological sub-health group had a low SC fluctuation variance and significant healing effect when they conducted a virtual immersive experience in the scene after ecological landscape design. This is consistent with the research results of Wu et al. (2022), Palomba et al. (2000), Ward et al. (2003) and Drachen et al. (2011) [24,25,27]. In the later stage, VR technology can also be used to assist medical institutions to improve the negative emotions of psychological and physical sub-health groups with a history of mental sub-health diseases, and broaden the auxiliary treatment methods for patients with complex sub-health diseases. Secondly, with the normalization of the COVID-19 epidemic, socially vulnerable groups can immerse themselves in 3D roaming landscape scenes without leaving their homes, which can play a role in relaxing their body and mind and relieving pressure. Such 3D roaming technology can also simulate the construction site at the same time and reasonably arrange the construction schedule, which can effectively improve construction safety, shorten the project construction time and save construction costs. Finally, engineers can simulate the design scheme, edit the landscape design elements in real-time and adjust the 3D space effect through 3D roaming technology. It can also be applied and extended to the teaching practice of landscape design majors to deepen students’ understanding of the concept of space and exercise their practical ability.

Admittedly, this research has certain limitations. Since the operation proficiency of the handle serves as a control variable, firstly, the sense of vertigo and mobile sensitivity might affect the accuracy of the experimental data to a certain extent. In the pre-experiment stage, a pilot study can be carried out to help subjects perceive the experiment environment and familiarize handle operating in advance, to improve the accuracy of the experimental data. Our modeling approach is limited to the tools (e.g., Unity2019 and ErgoLAB platform) and materials (surfaces, structures, colors) we employed, the 3D scenes included may not represent all virtual landscape scenarios. Secondly, only 80 subjects were invited to the experiment, so the sample size can be expanded. Finally, the research on the effect of landscape healing proposed in this paper is only applicable to the psychological sub-health group to relieve the psychological sub-health symptoms such as stress, anxiety and depression, and does not involve other treatment methods.

## Figures and Tables

**Table 1:** Statistical results of the correlation between level of interests and stress emotion.

Category of Area of Interest	*Sig*	*p*	*n*
Vegetation landscape	0.038	<0.050	73
Flower landscape	0.008	<0.050	73
Building landscape	0.298	>0.050	73
Other landscapes	0.729	>0.050	73

## Data Availability

Not applicable.

## References

[1] Xue Y., Xu J., Liu G.H., Huang C., Feng Y.F., Xu M.Y., Cheng X.M. (2021). Evaluation of Sub-Health Status of Chinese Urban Residents Using the Sub-health Measurement Scale Version 1.0. Chin. Gen. Pract..

[2] Tan Y. (2016). Mental sub-health that can not be ignored. Labor. Secur. World.

[3] Zeng Z.X., Liu L. (2008). Correlative Study of Mental Health State and Fatigue Degree in Patients with Chronic Fatigue Syndrome. Chin. Gen. Pract..

[4] Dong R., Shao G., Liu X., Zhao J.Z. (2016). Landsenses ecology and ecological planning toward sustainable development. Int. J. Sustain. Dev. World Ecol..

[5] Ulrich R. (1984). View through a window may influence recovery from surgery. Science.

[6] Kaplan S. (1995). The restorative benefits of nature: Toward an integrative framework. J. Environ. Psychol..

[7] Kaplan R., Kaplan S. (1989). The Experience of Nature: A Psychological Perspective.

[8] Kellert S., Wilson E.O., Kellert S.R. (1995). Biophilia Hypothesis.

[9] Huisman E., Morales E., Hoof J.V., Kort H. (2012). Healing environment: A review of the impact of physical environmental factors on users. Build. Environ..

[10] Stichler J.F. (2001). Creating healing environments in critical care units. Crit. Care Nurs. Q..

[11] Kalantari S. (2014). Understanding healing environments: Effects of physical environmental stimuli on patients’ health and well-being. Herd.

[12] Hu G.Q., Ma L.H. (2017). Application of VR and AR in Wisdom Library. Libr. Work. Study.

[13] Zhang N., Lian J. (2020). Interactive architecture and garden landscape roaming design based on 3DMAX. Mod. Electron. Technique..

[14] Tan Y.L., Jia J.Y., Peng S., Huang A.M., Li G.Y. (2014). Survey on Some Key Technologies of Virtual Tourism System Based on Web 3D. J. Syst. Simulation.

[15] Kim Y.S., Hong S.H., Ok C.Y. (2007). Analysis of landscape information and web gis implementation of using 3d topographic modeling. J. Korea Contents Assoc..

[16] Lindquist M., Lange E., Kang J. (2016). From 3d landscape visualization to environmental simulation: The contribution of sound to the perception of virtual environments. Landsc. Urban Plan..

[17] Bennett R., Zielinski D.J., Kopper R. Comparison of interactive environments for the archaeological exploration of 3D landscape data. Proceedings of the IEEE Vis International Workshop on 3DVis.

[18] Choi H.S., Kim S.H. (2016). A content service deployment plan for metaverse museum exhibitions—centering on the combination of beacons and hmds. Int. J. Inf. Manag..

[19] Zhang F., Dai G., Peng X. (2016). A survey on human-computer interaction in virtual reality. Sci. Sin. Inf..

[20] Xu L.Q., Hu Y.Z. (2020). Healing Street a New Model of Healthy Street. Time Archit..

[21] Niki K., Okamoto Y., Maeda I., Mori I., Ishii R., Matsuda Y. (2019). A novel palliative care approach using virtual reality for improving various symptoms of terminal cancer patients: A preliminary prospective, multicenter study. J. Palliat. Med..

[22] Moscato S., Sichi V., Giannelli A., Palumbo P., Ostan R., Varani S., Pannuti R., Chiari L. (2021). Virtual reality in hane palliative care: Brief report on the effect on cancer-related symptomatology. Front. Psychol..

[23] Kabir M., Rice J.L., Bush S.H., Lawlor P.G., Hackbusch R. (2020). A mixed-methods pilot study of lifeview audiovisual technology: Virtual travel to support well-being and quality of life in palliative and end-of-life care patients. Palliat. Med..

[24] Wu Y., Zhang H.C., Wang N.N., Zhang Y.X. (2022). Virtual Reality Technology in the Management of Negative Emotions in Cancer Patients. Nurs. J. Chin. People’s Lib. Army.

[25] Palomba D., Sarlo M., Angrilli A., Mini A., Stegagno L. (2000). Cardiac responses associated with affective processing of unpleasant film stimuli. Int. J. Psychophysiol..

[26] Ward R., Marsden P. (2003). Physiological responses to different WEB page designs. Int. J. Hum. Comput. Stud..

[27] Drachen A., Yannakakis G., Nacke L.E., Pedersen A.L. Correlation between Heart Rate, Electrodermal Activity and Player Experience in First-Person Shooter Games. Proceedings of the 5th ACM Siggraph.

[28] Li H., Dong W., Wang Z., Chen N., Wu J., Wang G. (2021). Effect of a virtual reality-based restorative environment on the emotional and cognitive recovery of individuals with mild-to-moderate anxiety and depression. Int. J. Environ. Res. Public Health..

[29] Stenling A., Ivarsson A., Lindwall M., Gucciardi D.F. (2018). Exploring longitudinal measurement invariance and the continuum hypothesis in the swedish version of the behavioral regulation in sport questionnaire (brsq): An exploratory structural equation modeling approach. Psychol. Sport Exerc..

[30] Chen X.H., Li Y.Y., Song L.J., Wang Y.J. (2022). Theoretical Framework and Research Prospect of Digital Economy. J. Manag. World.

[31] Xu L., Meng R., Huang S., Chen Z. (2019). Healing oriented street design: Experimental explorations via virtual reality. Urban Plan. Int..

[32] Da L.J., Zhao C.F., Dan Y. (2022). The Impact of Implicit Theories of Health on People’s Mental Health During the COVID-19 Pandemic. Chin. J. Clin. Psychol..

[33] Kosydar-Bochenek J., Krupa S., Religa D., Friganović A., Oomen B., Brioni E., Iordanou S., Suchoparski M., Knap M., Mędrzycka-Dąbrowska W. (2022). The Perception of the Patient Safety Climate by Health Professionals during the COVID-19 Pandemic—International Research. Int. J. Environ. Res. Public Health.

[34] Lütke Lanfer S.S., Pfeifer R., Lahmann C., Wünsch A. (2022). How to Measure the Mental Health of Teachers? Psychometric Properties of the GHQ-12 in a Large Sample of German Teachers. Int. J. Environ. Res. Public Health.

[35] Laher Z., Robertson N., Harrad-Hyde F., Jones C.R. (2022). Prevalence, Predictors, and Experience of Moral Suffering in Nursing and Care Home Staff during the COVID-19 Pandemic: A Mixed-Methods Systematic Review. Int. J. Environ. Res. Public Health.

[36] Watson D., Clark L.A., Tellegen A. (1988). Development and Validation of Brief Measures of Positive and Negative Aflect:The PANAS Scales. J. Personal. Soc. Psychol..

[37] Huang L., Yang T.Z., Ji Z.M. (2003). Applicability of the Positive and Negative Affect Scale in Chinese. Chin. Ment. Health J..

[38] Wang X., Yuan Y., Jin Y. (2016). Research on Readers’ Interests in Patent Literature and Its Topology Representation. Libr. Inf. Serv..

[39] Scheirer J., Fernandez R., Klein J. (2002). Frustrating the user on purpose: A step toward building an affective computer. Interact. Comput..

[40] Khalfa S., Isabelle P., Jean-Pierre B., Manon R. (2002). Event-related skin conductance responses to musical emotions in humans. Neurosci. Lett..

[41] Liu Y., Hu M., Zhao B. (2019). Interactions between forest landscape elements and eye movement behavior under audio-visual integrated conditions. J. For. Res..

[42] Pei W., Guo X., Lo T. Detecting Virtual Perception Based on Multi-Dimensional Biofeedback a Method to Pre-Evaluate Architectural Design Objectives. Proceedings of the 26th CAADRIA Conference.

[43] Lin Z., Wang Y., Ye X., Wan Y., Lu T., Han Y. (2022). Effects of Low-Carbon Visualizations in Landscape Design Based on Virtual Eye-Movement Behavior Preference. Land.

[44] Song Y., Ning H., Ye X., Chandana D., Wang S. (2022). Analyze the usage of urban greenways through social media images and computer vision. Environ. Plan. B Urban Anal. City Sci..

[45] Song Y., Newman G., Huang X., Ye X. (2022). Factors influencing long-term city park visitations for mid-sized US cities: A big data study using smartphone user mobility. Sustain. Cities Soc..

[46] Fernandez J., Song Y., Padua M., Liu P. (2022). A Framework for Urban Parks: Using Social Media Data to Assess Bryant Park, New York. Landsc. J..

[47] Zhang C., Han W., Wang C. (2021). Effects of Urban Riparian Plants’ Color on Visual Fatigue. J. Chin. Urban For..

[48] Chen C.L., Wu F., Ma Y.M., Wei Y., Yang R.T. (2020). Study of the effect on empty-nesters’ physical and mental health of flower arrangement activity. J. Northwest Univ..

[49] Li D., Zhang Q. (2017). Preliminary study of Chinese traditional flowers and horticultural therapy. J. Beijing For. Univ..

[50] Schimmack U. (2001). Pleasure displeasure and mixed feelings: Are semantic opposites mutually exclusive?. Cogn. Emot..

[51] Wang L., Li Z., Liu H., Du W. (2007). Factor Structure of General Dimension Scales of Panas-Xin Chinese People. Chin. J. Clin. Psychol..

[52] Peng D.L. (2004). General Psychology.

[53] Ren J., Huang L., Zhang Z. (2012). Meditation Makes A Peaceful State of Mind: People’s Positive and Negative Emotional Response Can Be Reduced by Meditation Training. Acta Psychol. Sin..

[54] Gouizi K., Reguig F.B., Maaoui C. (2011). Emotion recognition from physiological signals. J. Med. Eng. Technol..

[55] Balconi M., Bortolotti A. (2012). Empathy in cooperative versus non-cooperative situations: The contribution of self-report measures and autonomic responses. Appl. Psychophysiol. Biofeedback.

[56] Balconi M., Canavesio Y. (2013). Emotional contagion and trait empathy in prosocial behavior in young people: The contribution of autonomic (facial feedback) and Balanced Emotional Empathy Scale (BEES) measures. J. Clin. Exp. Neuropsychol..

